# 云锡矿工肺癌危险因素的队列研究

**DOI:** 10.3779/j.issn.1009-3419.2013.04.03

**Published:** 2013-04-20

**Authors:** 晓美 刘, 亚光 范, 勇 姜, 剑 向, 继先 王, 志娟 孙, 冠华 任, 树祥 姚, 润生 常, 永成 赵, 友林 乔, 清华 周

**Affiliations:** 1 300192 天津，天津市分子核医学重点实验室，中国医学科学院放射医学研究所，北京协和医学院 Peking Union Medical College & Institute of Radiation Medicine, Chinese Academy of Medical Science, Tianjin 300192, China; 2 300052 天津，天津市肺癌转移与肿瘤微环境重点实验室，天津市肺癌研究所，天津医科大学总医院 Tianjin Lung Cancer Institute, Tianjin Medical University General Hospital, Tianjin Key Laboratory of Lung Cancer Metastasis and Tumor Microenvironment, Tianjin 300052, China; 3 100020 北京，中国医学科学院肿瘤研究所，北京协和医学院 Department of Cancer Epidemiology, Cancer Institute, Chinese Academy of Medical Sciences and Peking Union Medical College, Beijing 100021, China; 4 300384 天津，天津智赢时代科技有限公司 Tianjin Zhiyin Technology Co., Itd, Tianjin 300384, China; 5 650500 昆明，昆明医学院公共卫生学院 School of Public Health, Kunming Medical University, Kunming 650500, China; 6 661000 个旧，云南个旧市红河州第三人民医院 the Third People's Hospital of Honghe Prefecture in Gejiu City, Yunnan Province, Gejiu 661000, China

**Keywords:** 肺肿瘤, 氡, 吸烟, 交互作用, 队列研究, Lung neoplasms, Radon, Tobacco use, Joint association, Cohort study

## Abstract

**背景与目的:**

吸烟是肺癌的主要病因, 在矿工肺癌病因的研究中发现, 职业暴露因素也起重要作用。本研究旨在分析氡暴露、吸烟等危险因素对云锡矿工肺癌的影响, 为预防和控制肺癌高发提供科学依据。

**方法:**

利用前瞻性队列研究方法, 对云锡矿工肺癌高危人群暴露因素对肺癌死亡的影响进行*Cox*多因素分析; 分析矿工肺癌危险与初始氡暴露年龄及氡暴露率的关系, 分析不同吸烟、氡暴露水平下肺癌死亡的危险, 并对吸烟和累积氡暴露量之间的交互作用进行分析。

**结果:**

进入研究时的年龄、吸烟量、累积氡砷暴露、既往慢性支气管炎为云锡矿工肺癌的独立危险因素, 教育程度是矿工肺癌的保护性因素; 肺癌危险与氡暴露率间存在逆剂量率效应, 但与初始氡暴露年龄无明显关联; 吸烟和氡暴露对肺癌危险有显著的相加交互作用。

**结论:**

云锡矿工肺癌高死亡率是多种因素共同作用的结果, 危险因素间的交互作用值得进一步深入研究。

## Abstract (en)

**Background and objective:**

Smoking is a major cause of lung cancer.Studies of lung cancer among miners have shown that occupational exposure also played an important role.The aim of this study is to investigate radon, cigarette use and other risk factors of lung cancer in Yunnan tin miners and to provide a scientific basis for the prevention and control of occupational lung cancer.

**Methods:**

A prospective cohort study was conducted among Yunnan tin miners, the associations between potential risk factors for lung cancer were analyzed by multivariate *Cox* regression model.Effects of age at first radon exposure and radon exposure rate on lung cancer risk were analyzed.The relationship between cumulative working level month and lung cancer was analyzed according to smoking status.The joint effect of tobacco use and cumulative radon exposure was analyzed based on additive and multiplicative models.

**Results:**

Increased risk of lung cancer was associated with age at enrollment, tobacco use, prior bronchitis, and cumulative arsenic and radon exposure, while higher education level was associated with decreased lung cancer risk.An inverse effect of radon exposure rate was observed.There was no significant association between lung cancer risk and first radon exposure age.There was a significant additive interaction between tobacco use and radon exposure on lung cancer risk.

**Conclusion:**

Several risk factors may contribute to the high incidence of lung cancer in Yunnan tin miners.Further studies are warranted to evaluate joint effect of different risk factors.

肺癌是世界范围内首要癌症死亡原因^[1]^。依据第3次全国恶性肿瘤回顾性死因抽样调查结果, 我国肺癌死亡率居恶性肿瘤死亡率首位(男性和女性年龄标化死亡率分别为39.1/10^5^、16.7/10^5^)^[2]^。自20世纪70年代发现我国云南锡矿矿工(下文简称云锡矿)肺癌发病率、死亡率高且已被确认为职业癌。姚树祥等^[3]^研究发现, 在1954年-2002年间, 云锡矿男性矿工肺癌年平均发病率为187.7/10^5^, 而有30年以上井下工作史人群的发病率则高达1, 694.9/10^5^, 年平均死亡率为161.0/10^5^, 远高于当地普通男性居民肺癌标化死亡率(26.6/10^5^)。

吸烟被公认为肺癌首要危险因素, 此外环境烟草烟雾、氡、职业暴露等其它因素也增加肺癌发病危险^[4-6]^。研究^[7]^已证明云锡矿工肺癌死亡率高与生产环境中的氡、砷等职业暴露因素有关。本研究拟利用云锡矿工职业高危人群前瞻性队列研究资料, 探讨云锡矿工肺癌危险因素及交互作用。

## 1 对象与方法

### 1.1 研究人群

研究对象来自于1992年-1999年参加“云锡矿工肺癌早期标志物”队列研究的成员, 其纳入标准为:①年龄为40岁及以上; ②至少有10年井下工作史或冶炼史或两者相加满10年; ③无恶性肿瘤史; ④知情同意。本研究中, 研究对象至少参加过1次1992年-1999年年度性肺癌筛查。

### 1.2 肺癌病例的确认和死亡

1992年-1999年, 每年对队列成员进行一次痰细胞学和X线胸片筛查。肺癌病例确认途径主要为:①肺癌筛查阳性者住院进一步检查后确诊; ②因症状于云锡总医院就医而确诊; ③根据云锡肿瘤登记系统提供的资料确诊; ④个别病例根据随访结果死因推断。

### 1.3 暴露情况

#### 1.3.1 累积氡暴露量资料

根据云锡矿井作业环境中氡监测浓度, 按照国际通用计算方法计算。累积氡暴露量的常用单位为工作水平月(working level month, WLM), 1 WLM相当于在1 WL下暴露1个工作月(每年工作2, 000 h/12 m≈170 h)^[8]^。

累积氡暴露量计算:按照氡暴露健康效应委员会(Committee on Health Effects of Exposure to Radon, BEIR Ⅵ)给出的暴露量-年龄-暴露持续时间(exposure-age-duration, EAD)模型计算^[9]^(暴露截止时间为1995年12月31日)。模型表达式如下所示:

*RR*(*a*)=1+*β*(*W*_5-14_+*θ*_15-24_*W*_15-24_+*θ*_25_+*W*_25+_)*Φ*_age_*γ*_z_

*W*_5-14_、*W*_15-24_、*W*_25+_分别表示发病前5年-14年、15年-24年及≥25年间的累积氡暴露量, *θ*_15-24_、*θ*_25+_表示发病前15年-24年及≥25年间氡暴露危险的相对贡献。其具体参数为:*θ_5-14_*=1.00, *θ*_15-24_=0.72, *θ*_25+_=0.44。累积氡暴露量*W*(WLM)=*W*_5-14_+*θ*_15-24_*W*_15-24_+*θ*_25+_*W*_25+_。

#### 1.3.2 氡暴露率

氡暴露率计算方法有多种, 本文的计算方法为:肺癌潜伏期外的累积氡暴露量/潜伏期外的暴露时间(以年为单位)。

#### 1.3.3 砷暴露量

以砷暴露指数(index of arsenic exposure months, IAEM)估算矿工砷暴露量, 计算方法:按照不同年代作业环境中砷浓度(mg/m^3^)与累积月之乘积做累加。

#### 1.3.4 吸烟量

用吸烟指数(包/日*年, pack-years)表示, 为每天吸纸烟包数乘以吸烟年数。对于旱烟或水烟, 按每1.25 g旱烟或水烟为一支纸烟计算^[10]^。

#### 1.3.5 年龄、性别、教育水平、既往肺部疾病史

上述信息均在研究对象进入队列时填写基线调查表获得。既往肺部疾病由医生询问获得, 此外, 矽肺必须有云锡劳动保护研究所开具尘肺诊断证明书才能确认。具体分组情况见表 1。

**1:** 云锡矿工队列人群特征及肺癌死亡分布 Characteristics and lung cancer mortality of Yunnan tin miner cohort

Characteristics	Participants No.	Person-year	Case No.	Death rate	Death rate ratio (95%CI)
Age (yr)					
< 60	6, 169	49, 707.9	107	215.3	1.00
60-69	2, 494	19, 699.0	256	1, 299.6	6.01 (4.80-7.53)
≥70	632	4, 139.2	80	1, 932.7	9.36 (7.00-12.51)
Gender					
Female	599	4, 485.6	2	44.6	1.00
Male	8, 696	69, 060.5	441	638.6	13.85 (3.45-55.58)
Education level					
No	2, 206	17, 390.1	222	1, 276.6	1.00
≤6 y	4, 457	36, 519.1	198	542.2	0.42 (0.35-0.51)
＞6 y	2, 632	19, 636.9	23	117.1	0.09 (0.06-015)
Smoking status					
Never	1, 451	11, 148.7	19	170.4	1.00
Former	894	6, 918.0	53	766.1	3.85 (2.42-6.11)
Current	6, 950	55, 479.4	371	668.7	4.40 (2.60-7.43)
Pack-years of smoking					
Q1 (0)	1, 451	11, 182.1	19	169.9	1.00
Q2 (1-583)	1, 968	15, 565.1	117	751.7	4.33 (2.66-7.03)
Q3 (584-1, 022)	1, 961	15, 674.1	129	823.0	4.74 (2.93-7.67)
Q4 (1, 023-1, 540)	2, 006	16, 228.8	103	634.7	3.65 (2.24-5.96)
Q5 (> 1, 540)	1, 909	14, 896.0	75	503.5	2.93 (1.77-4.85)
Cumulative arsenic exposure (IAEM)					
Q1 (0-1, 455)	2, 440	18, 444.7	13	70.5	1.00
Q2 (1, 456-7, 102)	2, 284	19, 394.7	75	386.7	5.27 (2.92-9.50)
Q3 (7, 102-17, 435)	2, 286	18, 180.6	242	1, 331.1	18.41 (10.54-32.17)
Q4 (≥17, 435)	2, 285	17, 526.1	113	644.8	9.08 (5.11-16.12)
Previous asthma					
No	8, 620	68, 337.0	377	551.7	1.00
Yes	675	5, 209.1	66	1, 267.0	2.29 (1.76-2.97)
Previous chronic bronchitis					
No	6, 866	54, 226.7	244	450.0	1.00
Yes	2, 429	19, 319.4	199	1, 030.1	2.26 (1.88-2.73)
Previous silicosis					
No	8, 841	70, 125.4	389	554.7	1.00
Yes	454	3, 420.7	54	1, 578.6	2.86 (2.15-3.80)
Previous tuberculosis					
No	9, 031	71, 435.0	425	594.9	1.00
Yes	264	2, 111.1	18	852.6	1.14 (0.88-2.26)
Cumulative radon exposure					
0	1, 846	14, 428.3	17	117.8	1.00
Q1 (0-95.82)	1, 865	14, 102.4	33	234.0	2.00 (1.12-3.59)
Q2 (95.82-148.44)	1, 860	15, 079.6	49	324.9	2.72 (1.57-4.73)
Q3 (148.44-295.84)	1, 862	15, 276.5	129	844.4	7.03 (4.24-11.66)
Q4 (≥295.84)	1, 862	14, 659.3	215	1, 466.6	12.30 (7.51-20.17)

### 1.4 统计学分析

计算不同人群特征和职业暴露肺癌死亡率, 利用*Cox*回归模型分析云锡矿工肺癌危险因素, 分析氡暴露人群中初始氡暴露年龄和暴露率对肺癌危险的影响, 分析不同吸烟、氡暴露水平下肺癌死亡的危险。基于相加模型和相乘模型分析吸烟和累积氡暴露量的交互作用:①在*Cox*回归模型中纳入乘积项分析吸烟和累积氡暴露量之间的相乘交互作用; ②利用Rothman提出的针对*Logistic*或*Cox*回归模型的三个评价相加交互作用的指标评价吸烟与累积氡暴露量的相加交互作用^[11]^, 分别为①相对超危险度比(relative excess risk due to interaction, RERI); ②归因比(attributable proportion due to interaction, AP); ③交互作用指数(synergy index, S)。三个指标及其95%CI的计算根据Li的方法^[12]^。如果两因素无相加交互作用, RERI和AP的可信区间应包含0, S的可信区间应包含1, 以双侧*P* < 0.05为差异有统计学意义。

## 2 结果

### 2.1 队列特征

1992年-1999年, 进入队列并参加过肺癌年度性筛查的云锡矿工共9, 295人, 截止到2001年12月31日, 确诊肺癌死亡病例443人。表 1显示不同人群特征和职业暴露肺癌死亡率, 从肺癌死亡率比可以看出, 年龄、性别、教育水平、吸烟、职业性氡砷暴露、既往肺疾病史均为肺癌的可能危险因素。表 2为云锡矿工肺癌危险因素的多因素*Cox*回归分析结果, 表明年龄、吸烟量、职业性氡砷暴露量、慢性支气管炎史是此队列人群的独立危险因素, 而教育是保护性因素。

**2:** 云锡矿工肺癌危险因素的多因素分析 Multiple analysis for lung cancer risk among Yunnan tin miners

Characteristics	HR (95%CI)^*^
Age (yr)	
< 60	1.00
60-69	2.30 (1.79-2.96)
≥70	3.20 (2.30-4.48)
Education level	
No	1.00
≤6 y	0.79 (0.85-0.96)
> 6 y	0.50 (0.31-0.80)
Previous chronic bronchitis	
No	1.00
Yes	1.54 (1.27-1.86)
Smoking (pack-years)	
Q (0)	1.00
Q2 (1-583)	1.25 (0.76-2.06)
Q3 (584-1, 022)	1.54 (0.94-2.52)
Q4 (1, 023-1, 540)	1.69 (1.03-2.78)
Q5 (> 1, 540)	1.63 (0.98-2.73)
Cumulative arsenic exposure (IAEM)	
Q1 (0-1, 455)	1.00
Q2 (1, 456-7, 102)	2.58 (1.40-4.79)
Q3 (7, 102-17, 435)	6.17 (3.31-11.52)
Q4 (≥17, 435)	6.53 (3.43-12.37)
Cumulative radon exposure	
0	1.00
Q1 (0-95.82)	2.88 (1.53-5.40)
Q2 (95.82-148.44)	4.02 (2.16-7.47)
Q3 (148.44-295.84)	5.06 (2.86-8.96)
Q4 (≥295.84)	4.95 (2.83-8.67)
^*^Adjusted for age, gender, education, smoking, cumulative radon exposure, cumulative arsenic exposure and previous lung disease. HR:hazard ratio.	

### 2.2 初始氡暴露年龄、氡暴露率与肺癌的关系

前瞻性队列人群中共9, 295人, 其中有氡暴露者7, 406人, 表 3显示了7, 406名氡暴露矿工肺癌危险与初始氡暴露年龄和氡暴露率的*Cox*多因素回归分析结果:在分析中, 当初始氡暴露年龄和氡暴露率为分类变量时, 初始氡暴露年龄、氡暴露率与肺癌无显著的剂量效应关系; 而当初始暴露年龄和氡暴露率为连续变量时, 氡暴露率是肺癌死亡危险的保护性因素, 调整肺癌死亡危险比(hazard ratio, HR)=0.98(0.96-0.99)。

**3:** 氡暴露云锡矿工肺癌危险与初始氡暴露年龄和氡暴露率的相关分析 Relationship between lung cancer risk and age at first radon exposure, rate of radon exposure among radon-exposed miners

Model	Characteristics	HR (95%CI)^*^
Model 1	Age at first radon exposure	
	Q1 (≤15)	1.00
	Q2 (16-19)	1.10 (0.85-1.42)
	Q3 (20-23)	1.23 (0.90-1.68)
	Q4 (≥24)	0.93 (0.66-1.32)
	Exposure rate (WLM/a)	
	Q1 (0-4.60)	1.00
	Q2 (4.60-6.66)	1.55 (1.05-2.27)
	Q3 (6.66-13.49)	1.18 (0.81-1.71)
	Q4 (> 13.49)	0.97 (0.64-1.45)
Model 2	Age at first radon exposure	0.99 (0.98-1.02)
	Exposure rate (WLM/a)	0.98 (0.96-0.99)
^*^Adjusted for age, gender, education, smoking, cumulative radon exposure, cumulative arsenic exposure and previous lung disease.		

### 2.3 不同吸烟、氡暴露水平下肺癌死亡的危险

由表 4可见, 相同的吸烟水平下, 肺癌的危险随累积氡暴露量的增大而增大, 在不吸烟者中, 低水平氡暴露、高水平氡暴露的HR分别为5.75(95%CI:1.26-26.12)和11.66(95%CI:3.24-42.05)。在相同的累积氡暴露水平内, 在高剂量组中肺癌的危险随吸烟量的增大而增大。与无吸烟和氡暴露组相比, 吸烟量 > 43.7 pys且氡暴露 > 118.44 WLM的HR高达17.15(95%CI:5.33-55.16)。

**4:** 按吸烟量分层后肺癌调整危险比与累积氡暴露量的相关分析 Relation of cumulative radon exposure to lung cancer risk by smoking

Smoking status	Cumulative radon exposure (WLM)	Adjusted HR (95%CI)	
Never	No	1.00	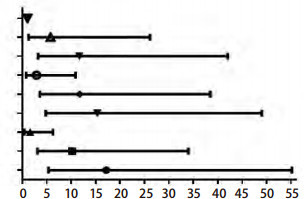
	≤118.44	5.75 (1.26-26.12)	
	> 118.44	11.66 (3.24-42.05)	
≤43.7 pks	No	2.89 (0.77-10.89)	
	≤118.44	11.73 (3.58-38.46)	
	> 118.44	15.32 (4.79-49.02)	
> 43.7 pys	No	1.56 (0.39-6.25)	
	≤118.44	10.19 (2.06-34.00)	
	> 118.44	17.15 (5.33-55.16)	

### 2.4 累积氡暴露量与吸烟对肺癌危险的交互作用分析

基于相加模型和相乘模型分析吸烟和累积氡暴露量的交互作用, 结果如表 5所示。在*Cox*回归模型中纳入乘积项分析吸烟和累积氡暴露量之间的相乘交互作用, 乘积项系数β为0.09(HR=1.10, 95%CI:0.57-2.12), 无统计学意义, 尚不能认为二者对肺癌危险存在单纯的相乘交互作用。

**5:** 累积氡暴露量和吸烟对肺癌危险的交互作用分析 Interaction of cumulative radon and smoking on lung cancer risk

Smoking	Cumulative radon (WLM)	Parameter *β*	HR (95%CI)^*^
≤25 pack-years	≤118.44	-	Reference
	> 118.44	0.84	2.32 (1.27-4.21)
> 25 pack-years	≤118.44	0.37	1.45 (0.79-2.68)
	> 118.44		3.69^**^
Multiplicative		0.09	1.10 (0.57-2.12)
Additive interaction	RERI		0.92 (0.11-1.73)
	AP		0.25 (-0.05-0.55)
	SI		1.52 (0.85-2.70)
^*^Adjusted for age, gender, education, smoking, cumulative radon exposure, cumulative arsenic exposure and previous lung disease; ^**^=e ^(0.84+0.37+0.09)^.			

利用Rothman提出的针对*Cox*回归模型的三个评价相加交互作用的指标评价吸烟与累积氡暴露量的相加交互作用, 结果如表 5所示, RERI、AP、S分别为0.92(0.11-1.73)、0.25(-0.05-0.55)和1.52(0.85-2.70), 其95%区间分别大于0、包括0、包括1, 说明累积氡暴露量与吸烟对云锡矿工肺癌的死亡有相加交互作用。图 1为累积氡暴露量与吸烟相加交互作用示意图。

**1:**
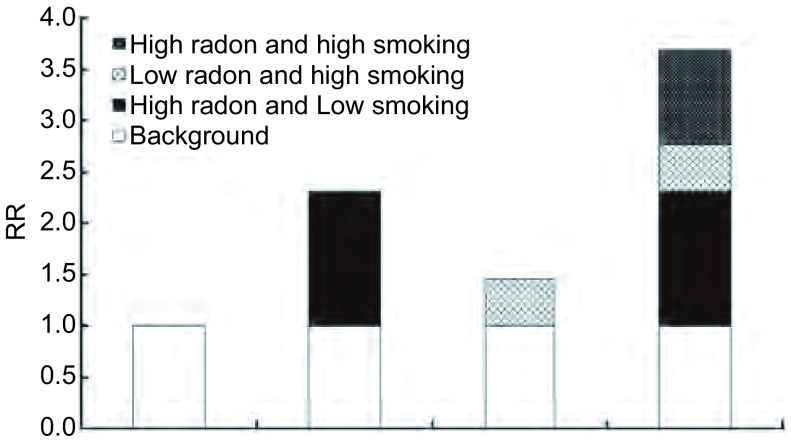
累积氡暴露量和吸烟交互作用示意图 Additive interaction of cumulative radon and smoking on lung cancer risk

## 3 讨论

国内外研究结果表明, 矿工肺癌发病与多种暴露因素有关。姚树祥等^[13]^曾报道云锡矿工的主要危险因素为职业性因素, 如氡、砷、粉尘等; 同时还与一些非职业性危险因素有关, 如吸烟、慢性支气管炎史、文化水平等。我们的研究除分析各危险因素对云锡矿工肺癌死亡危险的影响外, 还对初始氡暴露年龄、氡暴露率及吸烟和氡暴露交互作用对肺癌死亡危险的影响进行了分析。不同人群特征及职业暴露水平下肺癌死亡率的差别显示年龄、性别、文化水平、吸烟、职业性氡砷暴露、肺疾病既往史等因素与肺癌死亡有关, 继而多因素*Cox*回归分析结果显示, 年龄、慢性支气管炎史、吸烟、职业性氡砷暴露是肺癌的独立危险因素, 教育水平为保护性因素。

Xuan等^[14]^、Lubin等^[15]^研究发现肺癌危险与初始氡暴露年龄有关。本研究利用*Cox*回归模型分析了初始氡暴露年龄与肺癌危险的关系, 分别把初始氡暴露年龄按分层变量和连续性定量变量进行分析, 肺癌死亡危险与初始氡暴露年龄间均无统计学意义。其他研究^[16]^也有类似报道, 初始氡暴露年龄与肺癌危险的关系有待进一步深入研究。用同样的方法分析肺癌危险与氡暴露率的关系, 当暴露率为连续性变量时, 肺癌危险与氡暴露率之间存在逆剂量率效应(HR=0.98, 95%CI:0.96-0.99), 这与之前的报道结果一致^[17, 18]^。

本研究对吸烟和累积氡暴露量对肺癌危险的作用作了重点分析。吸烟被公认为是引起肺癌最重要的危险因素, 为分析累积氡暴露量对肺癌的影响, 同时排除吸烟的影响, 本研究在校正混杂因素后采用分层分析, 结果显示随累积氡暴露量的增加肺癌危险递增; 无氡暴露吸烟者肺癌危险要远小于有氡暴露不吸烟者。Taylor等^[19]^曾在研究中指出, 云锡矿工肺癌病因中, 砷暴露 < 吸烟 < 氡暴露, 本文结果与上述结果一致。为进一步研究吸烟和累积氡暴露对肺癌危险的交互作用, 本文又对二者之间的相加交互作用和相乘交互作用进行分析, 结果显示二者之间存在相加交互作用, 相乘交互作用无统计学意义。Xuan等^[14]^就吸烟者和非吸烟者的研究发现:吸烟和氡暴露存在介于相加和相乘作用之间的亚相乘作用, Lubin等^[20]^的研究也支持二者的关系介于相加和相乘模型之间。Breslow等^[21]^提出利用广义相对危险度模型分析因素间的包括相加、相乘的一系列交互作用形式, 本研究中仅对相加和相乘交互作用进行了分析, 我们将继续对吸烟和氡暴露的交互作用进行深入分析。

本研究的优点在于:累积氡暴露量的计算较以往的计算方法更科学; 研究为对云锡矿工队列的随访, 较以往云锡肺癌危险研究随访时间更长, 随访的肺癌病例更多, 此外职业性氡砷暴露、吸烟、肺疾病等信息是在基线调查中获得, 均在肺癌发病、死亡之前。缺点在于:云锡矿工肺癌危险因素较多, 在分析过程中尽管采用多因素分析方法, 但在分析过程中不能保证控制得当。

总之, 在研究职业性肺癌的危险因素时要注意危险因素种类的复杂性。通过本研究结果发现, 控制矿工肺癌的关键是控制肺癌危险因素, 通过通风除尘、湿式作业等途径降低作业环境中氡、砷以及粉尘的浓度; 控制吸烟等不良嗜好; 尽量减少职业性暴露机会; 严格控制矿工的纳入标准等措施的实施, 从而以最大的可能控制和降低职业性肺癌的发病率和死亡率。
